# Kidney Transplant Recipients Show Limited Lung Diffusion Capacity but Similar Hydrogen Peroxide Exhalation as Healthy Matched Volunteers: A Pilot Study

**DOI:** 10.3390/jcm12226964

**Published:** 2023-11-07

**Authors:** Piotr Jan Nowak, Łukasz Sokołowski, Paweł Meissner, Ewa Pawłowicz-Szlarska, Agata Sarniak, Anna Włodarczyk, Rafał Nikodem Wlazeł, Anna Prymont-Przymińska, Dariusz Nowak, Michał Nowicki

**Affiliations:** 1 Department of Nephrology, Hypertension and Kidney Transplantation, Medical University of Lodz, Pomorska 251, 92-213 Lodz, Poland; ewa.pawlowicz@umed.lodz.pl (E.P.-S.); nefro@wp.pl (M.N.); 2 Department of Obstetrics and Gynecology, Polish Mother’s Memorial Hospital Research Institute, Rzgowska 281/289, 93-338 Lodz, Poland; lukasz.sokolowski@iczmp.edu.pl; 3 University Laboratory of Blood Pressure Regulation and Function of the Autonomic Nervous System, Medical University of Lodz, Pomorska 251, 92-213 Lodz, Poland; 4 Department of Clinical Physiology, Medical University of Lodz, Mazowiecka 6/8, 92-215 Lodz, Poland; agata.sarniak@umed.lodz.pl (A.S.); anna.przyminska@umed.lodz.pl (A.P.-P.); dariusz.nowak@umed.lodz.pl (D.N.); 5 Department of Sleep Medicine and Metabolic Disorders, Medical University of Lodz, Mazowiecka 6/8, 92-215 Lodz, Poland; anna.wlodarczyk@umed.lodz.pl; 6 Department of Laboratory Diagnostics and Clinical Biochemistry, Medical University of Lodz, Pomorska 251, 92-213 Lodz, Poland; rafal.wlazel@umed.lodz.pl

**Keywords:** kidney transplantation, lung diffusing capacity, pulmonary function, exhaled breath condensate, hydrogen peroxide

## Abstract

Patients with end-stage chronic kidney disease show higher systemic oxidative stress and exhale more hydrogen peroxide (H_2_O_2_) than healthy controls. Kidney transplantation reduces oxidative stress and H_2_O_2_ production by blood polymorphonuclear leukocytes (PMNs). Kidney transplant recipients (KTRs) may be predisposed to an impairment of lung diffusing capacity due to chronic inflammation. Lung function and H_2_O_2_ concentration in the exhaled breath condensate (EBC) were compared in 20 KTRs with stable allograft function to 20 healthy matched controls. Serum interleukin eight (IL-8) and C-reactive protein (CRP), blood cell counts, and spirometry parameters did not differ between groups. However, KTRs showed lower total lung diffusing capacity for carbon monoxide, corrected for hemoglobin concentration (TLCO_c_), in comparison to healthy controls (92.1 ± 11.5% vs. 102.3 ± 11.9% of predicted, *p* = 0.009), but similar EBC H_2_O_2_ concentration (1.63 ± 0.52 vs. 1.77 ± 0.50 µmol/L, *p* = 0.30). The modality of pre-transplant renal replacement therapy had no effect on TLCO_c_ and EBC H_2_O_2_. TLCO_c_ did not correlate with time after transplantation. In this study, TLCO_c_ was less reduced in KTRs in comparison to previous reports. We suggest this fact and the non-elevated H_2_O_2_ exhalation exhibited by KTRs, may result perhaps from the evolution of the immunosuppressive therapy.

## 1. Introduction

Kidney disease is associated with systemic oxidative stress which can further augment renal damage and cardiovascular disease [1,2]. The production of reactive oxygen species (ROS) by a variety of enzymes (e.g., nicotinamide adenine dinucleotide phosphate oxidase (NADPH), xanthine oxidase, lipoxygenase) as well as a decreased activity of enzymatic (e.g., superoxide dismutase, catalase) and low molecular weight antioxidants (e.g., glutathione) result in an increased systemic activity of ROS in chronic kidney disease (CKD) [1,2]. Another, significant source of ROS are superoxide anions, which dismutate into H_2_O_2_ in the mitochondrial respiratory chain due to increased amounts of incompletely reduced oxygen [1]. The imbalance between oxidants and antioxidants is reflected by elevated plasma concentrations of markers of peroxidative damage to various biomolecules (e.g., malondialdehyde, F2-isoprostanes, 8-oxo-7,8-dihydro-2-deoxyguanosine, protein carbonyl groups) which have been reported in CKD patients not yet requiring dialysis [3,4,5]. Further deterioration of kidney function, requiring an initiation of dialysis, increases the intensity of oxidative stress and harmful peroxidative reactions in CKD patients [6,7]. The degree of bio-compatibility of the hemodialysis (HD) membranes, the characteristics of dialysis solutions, the loss of low molecular weight antioxidants into the dialysate, and the activation of PMNs together with persistent uremia, are the main factors responsible for the augmentation of oxidative stress and inflammatory processes during renal replacement therapy (RRT) [6,8,9]. Hydrogen peroxide (H_2_O_2_) is one of the most over-produced ROS in CKD patients [1,2]. Increased concentrations of H_2_O_2_ were reported in the urine, plasma, and exhaled breath condensate (EBC) of the analyzed CKD patients [10,11,12,13,14,15]. EBC is a composition of (A)—particles or droplets that are aerosolized from the airway lining fluid, (B)—distilled water that arises through condensation of water vapor from the airway surface, and (C)—water soluble volatiles that are exhaled and absorbed into the condensing breath [16]. Water contained in the EBC, originates from the vascular compartment. Through the presence of type P1 aquaporins, water is enabled to cross the endothelial and alveolar epithelial barrier [17,18,19]. Since H_2_O_2_ is permeable through the same aforementioned P1 aquaporins in cellular membranes like water, it could be recognized not only as an airway oxidative stress marker in the course of inflammatory lung diseases [20] but also for the case of systemic overproduction of H_2_O_2_. Although successful renal transplantation attenuates in general end-stage CKD-associated oxidative stress and the graft function is linked to the restoration of oxidant–antioxidant balance [6], new factors arise (including allogenic stimulation and new medications) which may counteract the effect of improved renal excretory function on ROS production in kidney transplant recipients (KTRs) [21]. Oxidative stress is implicated in lower airway damage involving alveolar walls and impairment of gas exchange [22]. There has been only one study reporting significant restrictive and obstructive abnormalities as well as decreased diffusing capacity for carbon monoxide in pulmonary function tests in KTRs [23]. It suggests chronic inflammatory processes involving the alveolar-capillary barrier in KTRs, which may be associated with enhanced ROS generation. To our best knowledge, there has not been a study reporting parameters of exhalation of H_2_O_2_ in these patients. Moreover, the above-mentioned study, was performed more than 20 years ago, and since then, progress in the management of KTRs has been made, which could have an impact on ROS generation and lung function. Therefore, we conducted extensive pulmonary function tests including lung diffusing capacity for carbon monoxide and measured the concentration of H_2_O_2_ in the EBC of KTRs and compared them with values obtained from healthy matched controls. The objective of our study was to investigate whether there are any differences in lung function and H_2_O_2_ exhalation between the two groups. Additionally, we aimed to discuss if these differences, if present, could be linked to a history of kidney transplantation. Moreover, these study groups were compared with respect to their plasma levels of IL-8 (a pro-inflammatory cytokine stimulating ROS production by PMNs) [24,25,26], CRP for the assessment of systemic inflammation [27], and neutrophil gelatinase associated lipocalin (NGAL) as a marker of renal injury and possibly allograft function [28].

## 2. Material and Methods

### 2.1. Studied Population

This is a prospective, a cohort, and an observational study which included 40 subjects, 20 kidney transplant recipients (all cadaveric donations), and 20 healthy matched controls (Table 1). The kidney transplant recipients (with stable graft function) were recruited from the Kidney Transplant Outpatient Department of our hospital, while the healthy control group, were all medical staff volunteers. The inclusion criteria were as follows: ages between 25 and 65 years, a body mass index between 18 kg/m^2^ and 35 kg/m^2^, and a written informed consent prior to the initiation of the study. The exclusion criteria, on the other hand were the following: current or a history of cigarette smoking within the last three years, alcohol and illicit drug abuse, the presence of any chronic respiratory disease, a history of occupational exposure to air pollutants, a history of acute infectious or inflammatory diseases, and a use of any vitamin or food supplement within the last 3 months. Any systemic pharmacological treatment within the last 3 months was an additional excluding criterion for healthy controls, whereas the time of transplantation less than 6 months or a change of immunosuppressive treatment within the last 3 months were additional exclusion criteria for the KTR patients. Allowable comorbidities included arterial hypertension, dyslipidemia, diabetes mellitus or post-transplant diabetes mellitus. KTRs were further divided into two subgroups that were dependent on pre-transplant modality of RRT (HD or peritoneal dialysis, PD) for the assessment of its potential influence. The study was conducted according to the Declaration of Helsinki. The above protocol was reviewed and approved by the local Ethics Committee. 

### 2.2. Study Design

The primary outcome measure for the study was the concentration of H_2_O_2_ in the EBC collected from all enrolled individuals. Secondary outcome measures included total lung diffusing capacity for carbon monoxide, corrected for hemoglobin concentration (TLCOc) and other lung function parameters as detailed in Section 2.6. Additionally, concentrations of IL-8 and NGAL in plasma were also included as secondary outcome measures.

### 2.3. Study Protocol

The study protocol included two visits conducted on the first and third day of the study. At the first visit, fasting venous blood was collected (10 mL into Becton Dickinson (Franklin Lakes, NJ, USA) vacutainer tubes with EDTA and Sarstedt AG (Nümbrecht, Germany) Monovette tubes with clotting factor Serum Gel S) for the determination of blood cell count, plasma IL-8, NGAL and serum creatinine, and CRP. Blood samples were centrifuged for 15 min (1000× *g*, 4 °C) and the obtained plasma or serum specimens were stored at −80 °C (up to 3 months), until IL-8 and NGAL measurement. The second study visit always started at 10:00 a.m. and included breath condensate (EBC) collection for around 45 min. Afterwards lung functions were measured between 11:00 a.m. and 12:00 with a Master-Laboratory Screen (Jaeger Toennies, Wuerzburg, Germany).

### 2.4. Collection of Exhaled Breath Condensate (EBC)

The collecting device consisted of plastic mouthpiece (with inspiratory and expiratory valves and saliva trap) connected to a plastic tube with a built in single use, replaceable, low resistance pneumotach headpiece of Lungtest 1000 spirometer (MES s.c. Cracow, Poland) working as air flow meter. The other end of the tube was connected to a glass Liebig cooler (collecting tube 55 cm length, internal diameter 10 mm, external jacket diameter 36 mm, Labmed, Lodz, Poland cat. no. 6010) placed in an upright position. The Liebig cooler was cooled with ethanol pumped in a closed circuit and its temperature was kept at −6 °C with Julabo F12-ED Refrigerated/Heating Circulator (JULABO GmbH, Seelbach, Germany). This temperature was the lowest one that allowed collection of liquid EBC in the sterile plastic tube covered with ice (Sarstedt, Nümbrecht, Germany, volume 13 mL, internal diameter 14 mm) mounted at the base of the Liebig tube cooler. Further decrease in ethanol temperature (e.g., −6.5 °C) resulted in congelation of EBC inside the Liebig cooled tube and hindered its collection (dripping into the Sarstedt tube). EBC collection started at 10:00 a.m. Subjects were asked to breathe easily through the mouthpiece, wear a nose clip, and rinse their mouth with distilled water just before and at 10 and 20 min of collection. The collection was terminated when total exhaled air flow reached 250 L (usually after 35 min) and average yield of EBC was 2.3 mL. The obtained EBC was mixed immediately with MIX solution containing horseradish peroxidase (HRP) and 4-hydroxyphenylacetic acid (4-HPAA) to start reactions being the basis of H_2_O_2_ measurement [29]. Thus, the process of freezing–thawing of EBC that may have stimulated H_2_O_2_ decomposition was avoided. Mouthpiece, plastic tube, and Liebig cooler were incubated in 5% H_2_O_2_ solution for 24 h then rinsed with distilled water and dried before the first usage. After each session of EBC collection, these parts were washed with detergent in an ultrasonic bath, then sterilized with Meliseptol (B. Braun Medical AG, Melsungen, Germany), rinsed with distilled water, and carefully dried.

### 2.5. Determination of H_2_O_2_ Concentration in Exhaled Breath Condensate (EBC)

Reagents: HRP type-II, 4-HPAA were purchased from Sigma (St. Louis, MO, USA). 30% H_2_O_2_ solution was from Chempur (Piekary Slaskie, Poland). All other reagents were obtained from POCH (Gliwice, Poland) and were of analytical grade. Standard solutions of H_2_O_2_ used for the execution of calibration curves were freshly prepared by appropriate dilution of 30% H_2_O_2_ in water; the concentration was confirmed by the measurement of absorbance at 240 nm, molar extinction coefficient of 43.6 mol^−1^ cm^−1^ [30]. Sterile deionized pyrogen-free water (freshly prepared, resistance > 18 MΩ/cm, HPLC H_2_O Purification System, USF Elga, Buckinghamshire, UK) was used throughout the study. MIX solution was prepared by adding 1 mL of 10 mmol/L 4-HPAA solution in 10 mmol/L PBS (pH = 7.4) and 10 µL of 10 mg/mL HRP solution in 10 mmol/L PBS (pH = 7.4) to 9 mL of 100 mmol/L PBS (pH = 7.4). Then the MIX solution was stored at −80 °C in 500 µL aliquots until use but no longer than 2 months. The concentration of H_2_O_2_ in EBC was measured according to the method of Brooks et al. [29]. Briefly, 350 µL of freshly collected EBC was mixed with 350 µL of MIX solution incubated for 15 min at 37 °C and then the 2,2′-dihydroxybiphenyl-5,5′-diacetate oxidation product of 4-HPAA as a measure of the amount of H_2_O_2_ was determined fluorimetrically using a Perkin Elmer Luminescence Spectrometer LS-50B (Norwalk, CT, USA). Excitation was at 295 nm (slit width 10 nm), emission was measured at 405 nm (slit width 15 nm), and the photomultiplier voltage was 700 V. Readings were expressed in μmol/L using the regression equation Y = (X − X_0_) × 0.009 + 0.0003 (where Y = concentration of H_2_O_2_ in EBC—µmol/L, X = intensity of emission expressed in arbitrary units; X_0_ = intensity of emission given by a reference sample containing 10 mmol/L phosphate buffered saline (PBS), with pH = 7.4 instead of EBC. An equation was obtained from the calibration curve (six H_2_O_2_ concentration points from 0.06 µmol/L to 2.0 µmol/L). All individual results were analyzed as means from triplicate measurements.

### 2.6. Lung Function Measurement

Lung functions were measured with a Master-Laboratory Screen (Jaeger Toennies, Wuerzburg, Germany) according to the American Thoracic Standards [31,32]. This involved (spirometry test) measurement of forced vital capacity (FVC) and forced expiratory volume in the first second (FEV1), determination (with a tracer gas—9.5% helium) of total lung capacity (TLC), residual volume (RV) and alveolar volume (AV), and measurement of total lung diffusing capacity for carbon monoxide (single breath method), corrected for hemoglobin (TLCOc) and total lung diffusing capacity and corrected for hemoglobin and alveolar volume (TLCOc/AV). All individual results were expressed as a percentage of the predicted value for patient’s height, sex, and age, which made their comparisons independent from these otherwise possible confounding factors [33,34].

### 2.7. Other Determinations

The complete blood count was determined by an automated hematology analyzer ABX Pentra DX120 (Horiba, Kyoto, Japan), which uses electric impedance and hydrodynamic flow cytometry for cell count and absorbance spectrophotometry with oxyhemoglobin determination. Concentrations of creatinine and CRP in serum were determined with enzymatic colorimetric assay and immunoturbidimetric assay, respectively, with an automated analyzer AUDXC700 (Beckman Coulter, Brea, CA, USA). Plasma concentrations of cyclosporine (CsA), tacrolimus, and everolimus were measured with the use of Cobas e601 (Roche Diagnostics, Rotkreuz, Switzerland) module automated analyzer (electrochemiluminescence technology for immunoassay analysis) within one week preceding the collection of EBC. Estimated glomerular filtration rate was calculated according to the CKD-EPI equation [35]. Concentrations of plasma IL-8 and NGAL were measured with ELISA technique (Human Interleukin-8 Elisa, BioVendor Laboratory Medicine Inc., Brno, Czech Republic, Cat. No.: RD194558200R and Human lipocalin-2/NGAL Elisa, BioVendor Laboratory Medicine Inc., Brno, Czech Republic, Cat. No.: RD191102200R, respectively) in the Central Scientific Laboratory of the Medical University of Lodz.

### 2.8. Statistical Analysis

All results were shown as mean ± SD, median with interquartile range (IQR). Normality of data distribution was tested with the Kolmogorov–Smirnov–Lilliefors test. For independent comparisons Mann–Whitney U test or unpaired *t*-test were used according to the data distribution. The Brown–Forsythe test for analysis of the equality of the group variances was used prior to the application of the unpaired *t*-test and all compared variances were equal. Correlations between variables were determined using Pearson’s *r* and Spearman’s *ρ* according to normality of variable distribution. *p*-value of <0.05 was considered significant. All calculations and analyses were performed with Statistica (data analysis software system), version 13 (TIBCO Software Inc., Palo Alto, CA, USA).

## 3. Results

### 3.1. Characteristics of the Studied Groups

Table 1 shows baseline characteristics of the KTR group and healthy controls. There were no differences between the groups in relation to age, body mass, body mass index, white blood cell count, and plasma concentrations of IL-8 and CRP. KTR subjects had significantly higher mean serum concentrations of creatinine and plasma NGAL, whereas eGFR was significantly lower than the corresponding values found in healthy controls, respectively. The causes of kidney failure were glomerular disease in five patients, IgA nephropathy in four, polycystic kidney disease in three, diabetic nephropathy in one, tubulointerstitial nephritis in one, and hypertensive nephrosclerosis in one. In five KTRs the cause of renal failure was idiopathic. The time since the transplantation occurred was 45.6 ± 32.1 months (median = 39.4; IQR = 43.8 with min 7.4 and max 121.7). Ten KTRs had a history of pre-transplant peritoneal dialysis, while nine had a history of pre-transplant hemodialysis. One patient underwent a preemptive kidney transplantation. In the vast majority of patients, post-transplant immunosuppression included a combination of tacrolimus, mycophenolate, and prednisone. Other treatment regimens are listed in Table 2 and Table 3. The numbers of patients receiving each immunosuppressive substance are provided in Table 4. Concomitant therapies included insulin (1 patient), sulfonylureas (2), metformin (1), β-blockers (18), calcium channel blockers (10), α-blockers (7), angiotensin converting enzyme inhibitors (ACEI) (8), aldosterone antagonist (4), centrally acting antihypertensive drugs (2), angiotensin receptor blocker (1), statins (8), proton pump inhibitors (7 patients, prophylaxis of steroid-induced gastritis).

### 3.2. Lung Function Parameters

Table 5 shows the results of lung function determination expressed as percentage of predicted (for patient’s height, sex, and age [33,34]) and the comparisons between groups. They did not differ in respect to the majority of parameters (FVC, FEV1, FEV1/FVC, TLC, RV and AV, TLCOc/AV), however FVC and FEV1 were close to being statistically significant. The mean of TLCOc in KTRs was lower by 10% (*p* = 0.009) than in healthy controls, (Table 5). Kidney transplant recipients treated with peritoneal dialysis before transplantation (PD-KTRs) had similar TLCOc and TLCOc/AV as those treated with hemodialysis (HD-KTRs) (Table 6).

### 3.3. Concentration of H_2_O_2_ in Exhaled Breath Condensate

**Figure 1:**
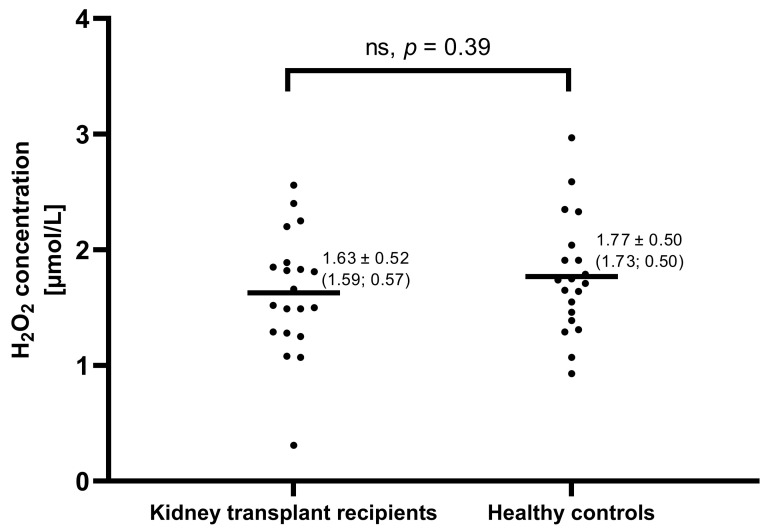
H_2_O_2_ levels in exhaled breath condensate of 20 kidney transplant recipients and 20 healthy matched controls. Each dot represents the result of one subject, whereas, each line, represents the mean value. Numerical results are expressed as mean ± SD (median; IQR). No significant differences were found between these groups.

The concentration of H_2_O_2_ in EBC in the KTR group was 1.63 ± 0.52 (1.59; 0.57) µmol/L and did not differ from healthy controls (Figure 1). No differences in EBC H_2_O_2_ between PD-KTR and HD-KTR subgroups were noted. Both PD-KTRs and HD-KTRs exhaled similar amounts of H_2_O_2_ as healthy controls (Table 6).

### 3.4. Correlations between the Concentration of Exhaled H_2_O_2_, Lung Diffusing Capacity, and Other Variables

In both groups, there were no significant associations between the H_2_O_2_ concentration in the EBC and concentrations of CRP, IL-8, NGAL, number of WBC and PMNS, as well as eGFR. Similarly, no significant associations were found between the EBC H_2_O_2_ and the TLCOc. Time after transplantation (equal to duration of immunosuppressive therapy) did not correlate with TLCOc (r = 0.09, *p* = 0.72) and TLCOc/AV (r = −0.1, *p* = 0.66).

## 4. Discussion

The study’s primary outcomes reveal that there were no significant differences in H_2_O_2_ concentration in EBC between KTRs and the control group. It is important to emphasize, however, that each tested group consisted of only 20 individuals, which is a rather limited number. Therefore, it should be recognized as a pilot study. When considering lung function outcomes, it was observed that only one out of the eight measured lung parameters, specifically TLCOc, showed a significant, albeit not substantial, reduction in the KTR group. A value of 80% or more of predicted TLCOc is recognized as the normal lung function [34]. Lower values, however, represent its clinically significant reduction which can be mild (79% to 60%), moderate (59% to 40%), or severe (lower than 40%) [34]. Four out of 20 KTRs (20%) had a clinically mild reduced TLCOc (Table 7), while all healthy controls presented with a normal TLCOc. However, when TLCOc related to AV was analyzed, only one patient had a clinically important but mild reduction of TLCOc/AV (Table 7).

In a previous study, which was conducted by Ewert et al. including 75 KTRs, as many as 57% of them had a clinically important reduction of TLCOc. The reduction was classified as mild in 27%, and moderate in 24% of their patients [23]. The total percentage of patients with a reduced TLCOc, exceeded 2-times those seen in our study. In regard to TLCOc/AV, the percentage was even higher (76% vs. 5% in our study).

The aforementioned study was conducted more than 20 years ago [23], and the progress in end-stage CKD therapy over that time may in part explain the difference between the results of these studies. We may speculate that the improvement of dialysis biocompatibility took place, and because the majority of KTRs were earlier exposed to this method of RRT, the length of exposure or the modality of dialysis could have had an influence on their lung function. In spite of this, Ewert et al. found no correlation between the duration of the pre-transplant dialysis period and post-transplant lung function [23]. We did not observe any impact of pre-transplant RRT modality (HD vs. PD) on lung function in our patients (Table 6) which remains in agreement with the findings of Ewert et al.

While the Ewert et al. study included 16.5% of current smokers, it lacked information regarding the past smoking habits of current non-smokers. The authors reported no significant differences in terms of pulmonary restriction, obstruction, or lung diffusion impairment between smokers and non-smokers. It is worth noting that the history of smoking was only associated with a reduced K_CO_, a diffusion parameter that is not adjusted for blood hemoglobin concentration [23], thus limiting its overall importance. Since the hemoglobin levels of KTRs can vary depending on graft function, we did not assess K_CO_ among our patients.

The other factors which can impact lung condition are uremia-related both chronic and generalized inflammation [36], and the expected effect of immunosuppressive drugs on them. In the prospective part of the study of Ewert et al., a gradual decrease in TLCOc was found in 36 KTRs after a follow-up median period of 22 months [23], which, in our opinion, may suggest the existence of a continuous inflammatory process, damaging the structures of the alveolar-capillary barrier in these subjects. On the contrary, our study did not reveal any correlation between the time after transplantation and the deterioration of lung function parameters. The study of Ewert et al. differed from our study in the choice of immunosuppressive therapy. Nearly 60% of patients in the study of Ewert et al., received a regimen of only two drugs: a corticosteroid combined with either CsA or azathioprine (AZT). The remaining 40%, received a triple therapy. None of their patients were treated with mycophenolate mofetil (MMF), mycophenolate sodium (MPA), or tacrolimus (TAC). In our study almost all patients (except for two) received a triple immunosuppression therapy (Table 2 and Table 3) and only three (15%) received CsA and two AZT (10%). MMF/MPA was a second agent in most patients (Table 4). Thus, a majority of KTRs (n = 17), received an immunosuppressant other than CsA and 16 received MMF/MPA as a part of their immunosuppressive regimen (Table 2 and Table 4).

Alveolar macrophages and PMNs located inside the alveoli (depending on their inflammatory activation), may produce ROS including H_2_O_2_ [37,38]. Phagocytes are one of the main intra-alveolar sources of H_2_O_2_ in the EBC, while circulating PMNs seem to be the main extra-alveolar source [39,40]. Due to the presence of aquaporins which are present in the alveolar-capillary barrier [17,19], H_2_O_2_ can diffuse from the blood into the alveolar and epithelial lining fluid and contribute to the exhaled amount of this oxidant [41]. Excessive amounts of ROS, including H_2_O_2,_ were proven to damage the lungs of rats and impair their perfusion in a concentration dependent manner [42]. An additional source of ROS is the endothelium, which produces significant amounts of H_2_O_2_, and is utilized as a signaling second messenger [43]. In comparison to the healthy control group, CKD patients that were never treated before with HD as well as those undergoing HD, exhibited higher concentrations of H_2_O_2_ in the blood. This was evaluated through chemiluminescence [44]. Consequently, end-stage CKD patients treated with HD, had higher concentrations of H_2_O_2_ in the EBC compared to healthy controls [13]. H_2_O_2_ exhalation by KTRs has not yet been analyzed in any other studies. Our studied group, with a stable graft function, had an approximately 2-times lower eGFR than the matched healthy group. It may suggest a partial persistence of uremic conditions predisposing to systemic oxidative stress. Surprisingly, however, KTRs did not exhale more H_2_O_2_ than healthy matched controls; the H_2_O_2_ concentration in the EBC was similar. The modality of dialysis therapy used before transplantation also seemed to have no effect on H_2_O_2_ exhalation. Jüttner et al. showed that the production of H_2_O_2_ by circulating PMNs in response to inflammatory stimuli was reported to decrease within 1 day after renal transplantation to values noted in healthy controls [45]. That effect was likely due to a recovery from uremia in the early post-transplant period, but, regarding quick onset, it could be the result of applied immunosuppression.

Modern immunosuppressive treatment rarely uses CsA in favor of TAC, and AZT in favor of MMF/MPA; all newer agents have the potential to exert weaker pro-oxidant effects. In vitro experiments demonstrated that both CsA and TAC (0.1 µmol/L each, 24 h of incubation), stimulated H_2_O_2_ production via the endothelial cells of the human umbilical vein in vitro, however, CsA exerted such an effect that was stronger than TAC [46]. Therefore, it was postulated that the calcineurin inhibitors, CsA in particular, may contribute to endothelial dysfunction [46] by enhancing vascular ROS production. MPA on the contrary (1 to 10 µmol/L, 24 h incubation), inhibited resting and phorbol myristate acetate-dependent (PMA—a potent activator of protein kinase C) endothelial H_2_O_2_ production under the same conditions [46]. Thus, MPA-related attenuation of the superoxide anion and H_2_O_2_ generation from the endothelium may decrease the risk of its damage [44]. MPA also inhibits H_2_O_2_ release from PMNs [46], diminishing resting and PMA-stimulated H_2_O_2_ production by those cells in vitro [46,47]. Moreover, MMF inhibited the production of peroxynitrite from macrophages [48] which are present inside the alveoli together with PMNs [49,50]. The inhibitory effect of MMF on these cells, may reduce ROS generation in the alveolar-capillary barrier and protect it from peroxidative damage. Our KTRs had similar levels of plasma IL-8 and CRP as the healthy control group, suggesting the absence of intensive systemic inflammation in response to the stimulation of phagocytes.

Patients with end-stage kidney disease may show an endothelial dysfunction which can partially result from excessive ROS generation [51,52]. The damage of endothelial glycocalyx is reflected by increased concentration of circulating syndecan-1 and hyaluronan [51]. As endothelial dysfunction is a generalized phenomenon, it possibly affects the pulmonary circulation which can impact the lung diffusing capacity. A recent study presented that elevated plasma concentrations of syndecan-1 and hyaluronan in patients with end-stage chronic kidney disease decreased three months after kidney transplantation, suggesting the regression of endothelial dysfunction [52]. Although, the authors did not reveal regimens of immunosuppression administered to their patients, one may assume that the therapy was mostly comprised of MMF/MPA and TAC since such regimens are currently the most common [53]. TAC is a slightly weaker inducer of endothelial oxidative stress than CsA [46]. Together with MMF/MPA, which is an inhibitor of endothelial ROS production [46,47], they may enhance endothelium recovery in response to post-transplantation reduction of uremia [52]. We have not found any data regarding a similar action of AZT. On the contrary, AZT exerted a potentially toxic effect on vascular endothelium by altering the intracellular nucleotide balance [54].

Interference with pulmonary circulation by inhibiting the formation of hypoxia-inducible factor 1 (HIF-1) in pulmonary tissue, and calcineurin activity in lungs in rat experiments are other unwanted effects of CsA [55]. Under physiological conditions, hypoxic pulmonary vasoconstriction is responsible for properly matching the ventilation to perfusion, in order to maintain optimal gas exchange through the alveolar-capillary barrier and arterial oxygenation [56]. CsA may potentially induce impairment of hypoxic pulmonary vasoconstriction and in consequence decrease lung diffusing capacity. On the other hand, TAC had the advantage of not inhibiting HIF-1 activity, as tested in cellular lines (including the endothelium) in vitro [57] but it could possibly interfere with the adaptation of the pulmonary circulation to hypoxia through the FK-binding protein-12 pathway [58]. This pathway, however, is probably less related to potential induction of undesirable inflammation in airways than HIF-1 suppression [59].

In the study of Ewert et al., immunosuppressive therapy differed from the one administered to our patients in part regarding the kind of applied calcineurin inhibitor and antiproliferative agent. The vast majority of our patients were taking the third drug, a corticosteroid (prednisone), while the majority in the above-mentioned study was not. Corticosteroids were reported to inhibit ROS release, including H_2_O_2_, by human neutrophils and vascular smooth muscle cells [60,61,62] as well as suppressed inflammatory stimulation of endothelium [63]. This factor could also add to the limitation of H_2_O_2_ exhalation, possible lung tissue protection, and improve lung function in our group. However, the small dose of 5 mg prednisone daily may only have had a small impact.

Nevertheless, no significant effect of kidney transplantation on the activities of blood catalase and glutathione peroxidase [45] supports our explanation that inhibition of H_2_O_2_ generation, but not enhanced decomposition by antioxidant enzymes, is responsible for normal H_2_O_2_ exhalation in KTRs.

All the above-mentioned facts support the combination of TAC and MMF which might have led to much smaller lung damage observed in our study compared to the previous report [23].

Available clinical data on the influence of certain immunosuppressants on lung function are limited to specific clinical conditions. Performed research covered patients after lung transplantation (LTx) or with certain autoimmune lung diseases. Therefore, those data can only be loosely tied into our observations. Nevertheless, metanalyses of randomized controlled trials, aiming to compare treatment with CsA and TAC after LTx, showed the superiority of TAC, which was expressed in the lower incidence of lymphocytic bronchitis and bronchiolitis obliterans syndrome (BOS) among patients treated with TAC versus CsA [64,65]. The difference in the incidence of acute rejection remained debatable [64,65]. A study on LTx patients with BOS, reported an arrest of lung function deterioration (measured with FEV1), after switching from CsA to TAC [66]. In another small study performed in a similar setting, the stabilization of FEV1 after conversion from CsA to TAC was also reported. This was accompanied by a tendency of a decrease in nitric oxide exhalation (a marker of inflammation) [67].

A treatment regime that consisted of either AZT or MMF, displayed similar pulmonary effects among patients with rheumatoid arthritis-associated interstitial lung disease [68] or chronic hypersensitivity pneumonitis [69,70]. However, in one of the latter studies, there was a tendency for fewer numbers of treatment-emergent adverse events on MMF [70]. Reports concerning LTx patients, with respect to the outcomes after AZT or MMF applications, were equivocal. Certain studies presented the equality of both agents as the elements of immunosuppressive regimens, in terms of the incidence of BOS, acute rejection, or patient survival [71,72]. Results of other similar studies in the same field, were more favorable to MMF, linking this medication to fewer occurrence rates of acute rejection episodes, [73,74] slower deterioration of FEV1 [73], or lower mortality among treated patients [75]. When the two immunosuppressive regimens, consisting either of CsA + AZA + prednisone or TAC + MMF + prednisone, were compared in LTx patients, it became apparent that the latter regimen was related to improve patient survival and lower the incidence of acute allograft rejection [76]. The same study, also found a trend towards improved pulmonary function in a group treated with TAC + MMF + prednisone compared to CsA + AZA + prednisone [76].

Data referring to the comparison of everolimus (EVE) with other immunosuppressants, with respect to pulmonary effects, are scarce. When LTx patients were treated with EVE or AZT, the former agent was found superior concerning its better efficacy, manifesting with better preservation of FEV1, lower incidence of BOS, or acute rejection [77], although, its tolerability was worse with a higher frequency of drug-related side effects (including pneumonia) [77]. In another study on LTx patients, treatment with EVE versus AZT, was shown to be more effective in the reduction of the percentage of CD4+ lymphocytes in bronchoalveolar lavage (BAL), as well as in a decreasing number of CD4+ lymphocytes and neutrophils in endobronchial biopsy specimens (EBB) [78]. AZT, on the contrary, displayed only the ability to reduce the percentage of total lymphocytes in BAL, though increasing the number of neutrophils in EBB [78].

The above-mentioned clinical observations, seem to support our hypothesis of possible better pulmonary outcomes when newer lines of immunosuppressants are used, namely TAC vs. CsA and EVE or MMF vs. AZT. Considering the different mechanisms of action of these agents, the different ways of modulation of immune response and the different impact on a variety of target cells can play a role in this matter. However, it is difficult to answer the question of whether KTRs with stable allograft function, who are on either cyclosporine and/or azathioprine with coexisting pulmonary issues—assuming the absence of contraindications to TAC, MMF or EVE—should have the treatment switched to the latter agents. One is supposed to take into account the fact that KTRs present a different clinical setting in comparison to lung transplant recipients or patients with autoimmune pulmonary diseases, who until now were the only groups under investigation in that regard. Nevertheless, if such circumstances occur, it may be reasonable to consider a change of therapy with an individualized approach to the problem.

The primary limitation of our study stems from the relatively small sample size of KTRs (n = 20); therefore, it can only be considered a pilot study. Another limitation is the unavailability of reliable information regarding the smoking habits of study participants more than 3 years before the study, which could potentially impact their lung function. Matching with the control group, elimination of important comorbidities, and extensive assessment of the lung function of patients, are the strengths of our study. Additionally, we were the first to measure the exhalation of hydrogen peroxide in KTRs.

## 5. Conclusions

We found that kidney transplant recipients with stable allograft function had lower lung diffusing capacity than healthy matched controls but did not exhale more H_2_O_2_. It did not seem to be related to the modality of dialysis prior to transplantation. The impairment of lung function was clearly of a lesser extent than that reported by other authors two decades ago. We may speculate that the progress in the choice of immunosuppressive therapy including the withdrawal of CsA and the introduction of MMF/MPA as an antiproliferative agent perhaps may be responsible for the observed difference. We would like to reiterate that the groups examined in this pilot study were of limited size, and we have exercised caution when drawing conclusions.

## Figures and Tables

**Table 1:** Characteristics of kidney transplant recipients and healthy controls.

Demographic/Clinical Variables	Kidney Transplant Recipients	Healthy Subjects	*p* Value
Number	20	20	NA
Sex M/F	9/11	10/10	NA
Age [years]	47.1 ± 10.7 (47.0; 17.0)	46.0 ± 11.5 (44.5; 20.8)	0.75
Body mass [kg]	72.3 ± 15.2 (64.5; 18.3)	74.7 ± 14.8 (78.0; 19.5)	0.45
Body mass index [kg/m^2^]	25.2 ± 3.6 (25.5; 4.7)	25.4 ± 3.2 (25.3; 4.1)	0.84
Serum creatinine [µmol/L]	142.0 ± 71.4 (119.0; 44.0)	75.2 ± 15.0 (74.5; 16.8)	**0.000001**
eGFR (CKD-EPI) [mL/min × 1.73 m^2^]	52.6 ± 20.6 (55.5; 16.8)	103.6 ± 17.2 (107.1; 24.4)	**0.00000001**
Erythrocytes [×10^6^/µL]	4.6 ± 0.7 (4.7; 0.9)	4.7 ± 0.4 (4.8; 0.6)	0.64
Hemoglobin [g/dL]	13.5 ± 2.0 (13.4; 3.2)	13.9 ± 1.2 (13.7; 1.7)	0.43
WBC [×10^3^/µL]	7.4 ± 2.5 (7.1; 2.9)	6.6 ± 3.5 (5.8; 1.2)	0.13
Neutrophils [×10^3^/µL]	4.6 ± 2.0 (4.3; 1.5)	3.6 ± 0.9 (3.6; 1.0)	0.09
Serum CRP [mg/L]	2.0 ± 1.7 (1.8; 1.5)	1.3 ± 0.7 (1.3; 1.0)	0.25
Plasma IL-8 [pg/mL]	0.54 ± 0.99 (0.21; 0.41)	0.95 ± 1.71 (0.42; 1.0)	0.45
Plasma NGAL [ng/mL]	81.1 ± 44.1 (69.3; 45.3)	44.0 ± 16.8 (40.7; 24.1)	**0.002**

NA, not applicable; M, male; F, female; WBC, white blood cells; CRP, C-reactive protein; CKD-EPI, Chronic Kidney Disease Epidemiology Collaboration equation; eGFR, estimated glomerular filtration rate; IL-8, interleukin eight NGAL, neutrophil gelatinase associated lipocalin. Data are shown as mean ± SD and (median; interquartile range). Bold values denote statistical significance at the *p* < 0.05 level.

**Table 2:** Immunosuppressive therapy, time since transplantation, and modality of RRT before transplantation in the group of 20 kidney transplant recipients.

Patient Number	Time Since Transplantation [Months]	Modality of RRT before Transplantation	CsA Plasma Concentration [ng/mL] *	Tacrolimus Plasma Concentration [ng/mL] *	Everolimus Plasma Concentration [ng/mL] *	MMF [mg/day] ‡	AZT [mg/Day]	Prednisone [mg/Day]
1	45.0	PD	NA	6.33	NA	1000	NA	5
2	19.6	HD	NA	6.40	NA	1000	NA	5
3	29.7	HD	NA	4.81	NA	1000	NA	5
4	8.8	HD	NA	6.84	0.70	NA	NA	5
5	106.1	NA	NA	5.82	NA	1000	NA	5
6	13.6	PD	NA	NA	3.70	2000	NA	5
7	64.4	HD	60.41	NA	NA	720 †	NA	5
8	63.0	HD	NA	6.40	NA	720 †	NA	5
9	17.1	PD	NA	6.00	NA	2000	NA	5
10	121.7	HD	89.07	NA	NA	NA	125	5
11	25.7	HD	NA	6.40	NA	1000	NA	5
12	33.8	PD	NA	4.50	NA	NA	150	5
13	55.4	PD	NA	6.90	NA	1000	NA	5
14	67.5	PD	NA	NA	7.50	NA	NA	5
15	7.4	PD	NA	6.86	NA	2000	NA	5
16	82.9	HD	86.58	NA	NA	1000	NA	5
17	23.5	PD	NA	NA	5.50	1000	NA	5
18	61.5	PD	NA	5.44	NA	1000	NA	NA
19	45.2	PD	NA	7.10	NA	1000	NA	5
20	19.1	HD	NA	7.60	NA	1000	NA	5

RRT, renal replacement therapy; MMF, mycophenolate mofetil; AZT, azathioprine; HD, hemodialysis; PD, peritoneal dialysis; *—plasma concentrations determined within one week preceding the collection of EBC; †—mycophenolate sodium instead of MMF; ‡—given daily dose was divided into two equal doses; NA, not applicable. In patient No 14 the immunosuppressive therapy included only everolimus with prednisone due to past history of leukopenia. Patient No 5 underwent preemptive transplantation.

**Table 3:** Number of KTRs receiving certain immunosuppressive regimen.

Immunosuppressive Regimen	Number of Patients
TAC + MMF/MPA + prednisone	11
TAC + MMF/MPA	1
TAC + EVE + prednisone	1
TAC + AZT + prednisone	1
EVE + MMF/MPA+ prednisone	2
EVE + prednisone	1
CsA + MMF + prednisone	2
CsA + AZT + prednisone	1

TAC, tacrolimus; MMF, mycophenolate mofetil; MPA, mycophenolate sodium; EVE, everolimus; AZT, azathioprine; CsA, cyclosporine A.

**Table 4:** Number of KTRs receiving certain immunosuppressive agent.

Active Substance	Number of Patients
prednisone	19
MMF/MPA	16
TAC	14
EVE	4
CsA	3
AZT	2

TAC, tacrolimus; MMF, mycophenolate mofetil; MPA, mycophenolate sodium; EVE, everolimus; AZT, azathioprine; CsA, cyclosporine A.

**Table 5:** Lung function parameters in kidney transplant recipients and healthy subjects.

Lung Function Parameter *	Kidney Transplant Recipients	Healthy Subjects	*p* Value
FVC	113.4 ± 18.3 (115.2; 20.2)	122.7 ± 14.1 (118.8; 19.6)	0.08
FEV1	106.7 ± 18.5 (110.5; 25.1)	116.3 ± 13.5 (112.1; 19.0)	0.07
FEV1/FVC	98.3 ± 5.1 (98.7; 5.7)	99.2 ± 6.6 (99.6; 5.7)	0.63
TLC	102.6 ± 9.8 (104.0; 10.3)	106.6 ± 10.9 (107.9; 12.1)	0.23
RV	98.1 ± 12.1 (97.9; 10.2)	94.6 ± 13.5 (92.5; 19.2)	0.39
AV	102.6 ±10.1 (104.3; 10.7)	106.6 ± 11.5 (108.3; 12.2)	0.25
TLCOc	92.1 ± 11.5 (94.2; 17.7)	102.3 ± 11.9 (101.7; 10.9)	**0.009**
TLCOc/AV	92.8 ± 12.4 (88.9; 15.8)	99.1 ± 11.3 (100.4; 18.2)	0.10

FVC, forced vital capacity; FEV1, forced expiratory volume in the first second; TLC, total lung capacity; RV, residual volume; AV, alveolar volume; TLCOc, total lung diffusing capacity for carbon monoxide corrected for hemoglobin concentration; TLCOc/AV, total lung diffusing capacity for carbon monoxide corrected for hemoglobin concentration and alveolar volume; *—results are expressed as percent of predicted value and shown as mean ± SD and (median; interquartile range). Bold values denote statistical significance at the *p* < 0.05 level.

**Table 6:** Comparison of concentrations of H_2_O_2_ in exhaled breath condensate (EBC), total lung diffusing capacity for carbon monoxide corrected for hemoglobin concentration (TLCOc), and total lung diffusing capacity for carbon monoxide corrected for hemoglobin concentration and alveolar volume (TLCOc/VA), in subgroups of kidney transplant recipients—treated with peritoneal dialysis before transplantation (PD-KTR) or treated with hemodialysis before transplantation (HD-KTR) and healthy controls.

Group/Subgroup	Compared Variables		
	H_2_O_2_ in EBC [µmol/L]	TLCOc *	TLCOc/VA *
PD-KTR (*n* = 10)	1.67 ± 0.43 (1.58; 0.53)	88.7 ± 10 (86.5; 15.7)	89.5 ± 9.1 (87.8; 12.9)
HD-KTR (*n* = 9) ^§^	1.56 ± 0.66 ^‡^ (1.52; 0.59)	95.0 ± 13.0 ^#^ (96.9; 21.1)	96.4 ± 15.6 ^#^ (89.2; 21.6)
Healthy controls (*n* = 20)	1.77 ± 0.50 (1.73; 0.50)	102.3 ± 11.9 (101.7; 10.9)	99.1 ± 11.3 (100.4; 18.2)

^#^* p* = 0.25, ^‡^* p* = 0.55 vs. corresponding values in PD-KTRs; *—results are expressed as percent of predicted value and shown as mean ± SD (median; interquartile range); ^§^—one patient underwent pre-emptive transplantation.

**Table 7:** Individual results of lung diffusing capacity and concentrations of H_2_O_2_ in exhaled breath condensate in the group of kidney transplant recipients and healthy controls.

Subject Number	Kidney Transplant Recipients			Healthy Controls		
	TLCOc *	TLCOc/VA *	H_2_O_2_ [µmol/L]	TLCOc *	TLCOc/VA *	H_2_O_2_ [µmol/L]
1	107.4	95.1	1.7	81.8	88.8	1.3
2	112.1	107.3	1.3	101.3	94.5	1.6
3	102.6	104.7	2.2	92.8	88.2	1.9
4	105.6	119.1	1.8	118.0	110.1	1.1
5	100.3	92.6	1.5	117.5	111.1	1.9
6	83.7	81.8	1.8	99.2	91.6	2.0
7	73.0	73.4	1.1	95.5	100.5	1.6
8	105.6	115.3	1.9	100.5	110.5	1.7
9	96.0	99.0	0.3	102.0	100.3	1.7
10	96.9	85.6	1.5	105.6	107.8	2.6
11	84.5	85.7	2.3	107.3	90.6	1.8
12	78.8	87.1	1.3	82.4	81.6	1.7
13	97.3	93.2	1.1	101.2	105.7	0.9
14	94.8	88.5	1.5	114.1	100.6	3.0
15	84.2	80.4	1.5	97.3	112.0	2.3
16	93.5	89.2	2.4	106.0	98.7	1.8
17	88.8	80.9	1.8	109.0	84.6	2.4
18	77.9	107.7	1.3	126.6	105.3	1.5
19	77.8	81.7	2.6	82.8	79.2	1.3
20	81.5	87.3	1.9	105.9	119.4	1.4

TLCOc, total lung diffusing capacity for carbon monoxide corrected for hemoglobin concentration; TLCOc/VA, total lung diffusing capacity for carbon monoxide corrected for hemoglobin concentration and alveolar volume; *—expressed as percent of predicted value.

## Data Availability

The data presented in this study especially individual results of H_2_O_2_ concentration in exhaled breath condensate and lung diffusing capacity for carbon monoxide as well as the immunosuppressive treatment regimen are shown in Table 2 and Table 7 in the manuscript.
